# First Evidence of Familial Transmission of Hereditary Gastrointestinal Polyposis Associated with Germline *APC* Variant in Jack Russell Terriers

**DOI:** 10.3390/vetsci10070439

**Published:** 2023-07-05

**Authors:** Wakana Yoneji, Kyoko Yoshizaki, Teruaki Hirota, Kensuke Yoneji, Ryutaro Yoshikawa, Takashi Mori, Hiroki Sakai, Akihiro Hirata

**Affiliations:** 1 Laboratory of Veterinary Pathology, Joint Department of Veterinary Medicine, Faculty of Applied Biological Sciences, Gifu University, 1-1 Yanagido, Gifu 501-1193, Japan; 2 Nara Animal Referral Clinic, 5-20-7 Mitsugarasu, Nara 631-0061, Japan; 3 Animal Medical Center, Faculty of Applied Biological Sciences, Gifu University, 1-1 Yanagido, Gifu 501-1193, Japan; 4 Laboratory of Veterinary Clinical Oncology, Joint Department of Veterinary Medicine, Faculty of Applied Biological Sciences, Gifu University, 1-1 Yanagido, Gifu 501-1193, Japan; 5 Center for One Medicine Innovative Translational Research (COMIT), Gifu University, Gifu 501-1193, Japan

**Keywords:** *adenomatous polyposis coli*, dogs, hereditary disease, hereditary tumor, gastrointestinal tumor, germline variant, familial adenomatous polyposis, family tree, familial transmission, Jack Russell terrier

## Abstract

**Simple Summary:**

Hereditary gastrointestinal (GI) polyposis in Jack Russell terriers (JRTs) is a recently discovered disease caused by an identical germline variant in the *adenomatous polyposis coli* (*APC*) gene. To date, intrafamilial transmission of this disease has not been demonstrated, mainly due to the difficulty in tracing the family members of household dogs. In this study, we found three unrelated JRT lineages in which hereditary GI polyposis was transmitted across successive generations in association with the germline *APC* variant.

**Abstract:**

Jack Russell terriers (JRTs) with gastrointestinal (GI) neoplastic polyps have been recently reported to harbor an identical germline variant in the *adenomatous polyposis coli* (*APC*) gene, c.[462_463delinsTT], in the heterozygous state, which indicates that this disease is an autosomal dominant hereditary disorder. Many individual cases of this disease have been observed in clinical practice; however, familial transmission has not been demonstrated due to the difficulty in tracing the family members of household dogs, especially after the disease’s onset in adulthood. Recently, we encountered two cases of GI polyposis in maternal half sisters. These two cases facilitated the identification of additional relatives spanning three generations, including parents, full and half siblings of the dam (aunt and uncle), littermate and non-littermate siblings, and a nephew. Genetic analysis revealed that 11 of the 14 examined JRTs in this family carried the heterozygous germline *APC* variant, and eight dogs with the variant already had a current and/or past medical history of GI neoplastic polyps. Some cases in the family showed significantly more severe disease phenotypes than those initially reported, suggesting that the severity of this disease can vary considerably among individuals. Moreover, familial aggregation of severe cases suggested that the genetic modifier involved in increasing severity may have been transmitted in this family in addition to the germline *APC* variant. Furthermore, in addition to this family, we reported two other families of JRTs affected by hereditary GI polyposis that consisted of five full and half siblings and a mother–daughter pair, respectively. These findings unequivocally establish the transgenerational transmission of hereditary GI polyposis associated with the germline *APC* variant in JRT lineages.

## 1. Introduction

Recent advances in genetic technology and clinical veterinary medicine have led to the identification of causative gene variants for many genetic diseases in dogs [1,2,3]. In dogs, breed predisposition often provides the first clue to discovering a novel hereditary disorder [1]. Based on daily clinical practice and pathological examination findings, despite the stable population of Jack Russell terriers (JRTs) in Japan [4], the incidence of gastrointestinal (GI) neoplastic polyps has been steadily increasing since the late 2000s [5,6,7]. The low incidence of GI epithelial tumors in dogs [8,9,10,11] has highlighted the breed-specific increase in JRTs [6,7]. Recently, JRTs with GI polyps have been proven to harbor an identical germline variant in the *adenomatous polyposis coli* (*APC*) gene c.[462_463delinsTT] (GenBank accession numbers: LC598892.1–LC598902.1 and LC600201.1–LC600209.1) in the heterozygous state, thus making this disease an autosomal dominant hereditary disorder [7]. Consistent with previous findings that mice homozygous for the germline *APC* variant allele died during embryonic development [12,13], no homozygotes have been observed in dogs.

In JRTs, this hereditary disease is characterized by the development of solitary and multiple polyps in the stomach and large intestine, with a predilection for the gastric antrum and rectum; multiple lesions can develop simultaneously at both locations [7,14]. Most lesions are histopathologically diagnosed as adenomas or adenocarcinomas [7,14]. Initial GI lesions can arise at variable ages, reportedly between 2 and above 10 years and at an average age of 7.7 years [7]. Early onset and frequent recurrence of GI polyps are distinct features of this cancer-prone disease [7]. The main symptom of GI polyps varies depending on their location. Vomiting is the most common clinical sign in dogs with gastric polyps. At the same time, bloody stool is the most common clinical sign in dogs with colorectal polyps [7,14]. Hereditary GI tumors have a far better prognosis than sporadic cases with 1- and 2-year survival rates of 100% [7]. However, they have a high lifelong risk of recurrence. Genetic testing permits a definitive diagnosis of this hereditary disease in JRTs [15], and the number of newly diagnosed cases has been increasing in clinical settings in Japan [14].

On the basis of similarities in the responsible gene and disease phenotype, this hereditary disease is considered the canine equivalent of familial adenomatous polyposis (FAP) in humans [16]. However, partly because of a lack of evidence for familial transmission of the disease in dogs, we tentatively named this disease “hereditary GI polyposis” rather than “familial GI polyposis” [7]. In humans, when germline variants likely associated with a certain disease are observed in patients, it is possible to examine their close relatives, such as parents and siblings, relatively easily to determine whether the disease is transmitted in association with the candidate DNA variant within the family. For example, while more than 3000 different germline *APC* variants causing FAP have been identified [17], novel pathogenic *APC* variants have still been discovered in recent studies in which examinations of family members were conventionally performed [18,19,20,21]. In contrast, it is challenging to trace the relatives of household dogs after disease onset. In particular, compared with the cases where hereditary diseases or traits are apparent at or shortly after birth [22,23,24,25], it is more difficult to prove familial transmission of adult-onset genetic diseases, such as hereditary GI polyposis. To overcome this difficulty and demonstrate familial transmission, a previous study examined the blood relationships of five affected JRTs using their pedigree certificates. However, it failed to find any close relatives [7]. A more recent study conducted a pedigree analysis, again with nine variant carriers newly detected in an epidemiological survey on the germline *APC* variant frequency in JRTs in Japan [26]. While a few carrier dogs descended from a common ancestor with an uncertain *APC* genotype were observed, no first-degree relatives, such as parents, offspring, or full siblings, could be observed even in a large-scale study involving nearly 800 JRTs [26]. Therefore, while a case-control study has determined the association of the *APC* variant and GI polyposis in JRTs [7], it still lacks the generally accepted standard for familial disorders.

This study provides the initial evidence demonstrating the transgenerational transmission of hereditary GI polyposis in JRTs associated with the identified germline *APC* variant.

## 2. Materials and Methods

### 2.1. Information on Examined Families

Three unrelated families involving 21 JRTs were investigated. The first family included 14 dogs across three generations. The second and third families comprised five paternal siblings and a mother–daughter pair, respectively. Of the dogs, some overlapped with those examined previously; dogs A in family 1 and T in family 3 were employed in case-control study [7], and dogs B and N in family 1 and R and S in family 2 were detected in epidemiological study [26].

### 2.2. Pedigree Analysis

Pedigree certificates for all 21 JRTs were collected with the cooperation of the dog owners and breeders. The certificates included information on three generations of ancestry and individual details, such as birthdays, sex, and coat color. The reverse side of the certificates contained information on the number of male and female littermates. All pedigrees were issued by one of the two major kennel clubs in Japan: the Japan Kennel Club or KC Japan.

### 2.3. Germline APC Variant Genotyping

The germline *APC* variant c.[462_463delinsTT] status of the JRTs was evaluated by PCR direct sequencing, as previously reported [7,15]. Genomic DNA was extracted from ethylenediaminetetraacetic acid-anticoagulated peripheral blood. PCR was performed to amplify a 385 bp fragment containing the variant site in the *APC* gene using the following primers: forward—5′-AGTCCCACCTTCAAAAATCC-3′ and reverse—5′-AGTCCCACCTTCAAAAATCC-3′. After PCR amplification, the purified PCR products were subjected to sequencing analysis using the ABI Prism 3500 Genetic Analyzer (Applied Biosystems, Foster City, CA, USA) and the Big Dye Terminator v3.1 Cycle Sequencing Kit (Thermo Fisher Scientific, Waltham, MA, USA).

### 2.4. Clinical Tests

For dogs referred to the Nara Animal Referral Clinic, several clinical tests, including blood tests, radiography, ultrasound, computed tomography (CT), and endoscopy, were performed by experienced clinical veterinarians (W.Y. and K.Yon.). CT was performed using a 16-slice multi-detector row helical CT unit (Aquilion Prime, Canon Medical Systems, Inc., Tochigi, Japan). During endoscopy, a narrow scope with a 6 mm outer diameter (Fujinon Advancia, Fujifilm, Tokyo, Japan) was used to examine the stomach, duodenum, and colorectum. Clinical data from a few cases (dogs D, E, F, G, K, M, O, P, and Q) were described in our previous study that focused on the clinical diagnosis of this disease [14].

### 2.5. Histopathological Analysis

Endoscopically or surgically resected samples were fixed in 10% neutral-buffered formalin, routinely processed, and embedded in paraffin. Sections were stained with hematoxylin and eosin and examined by a single pathologist certified by the Japanese College of Veterinary Pathologists (A.H.). The GI lesions were diagnosed according to the WHO Classification of Tumors of the Alimentary System of Domestic Animals [8]. When previous lesions were diagnosed at commercial laboratories, pathology reports were obtained from primary-care veterinarians or dog owners, if available.

## 3. Results

### 3.1. Family Structures, Germline APC Variant Status, and Medical History

A total of 21 dogs from three distinct, unrelated families were examined. The dogs were assigned alphabetical letters based on the order of birth within each respective family.

#### 3.1.1. Family 1

**Figure 1:**
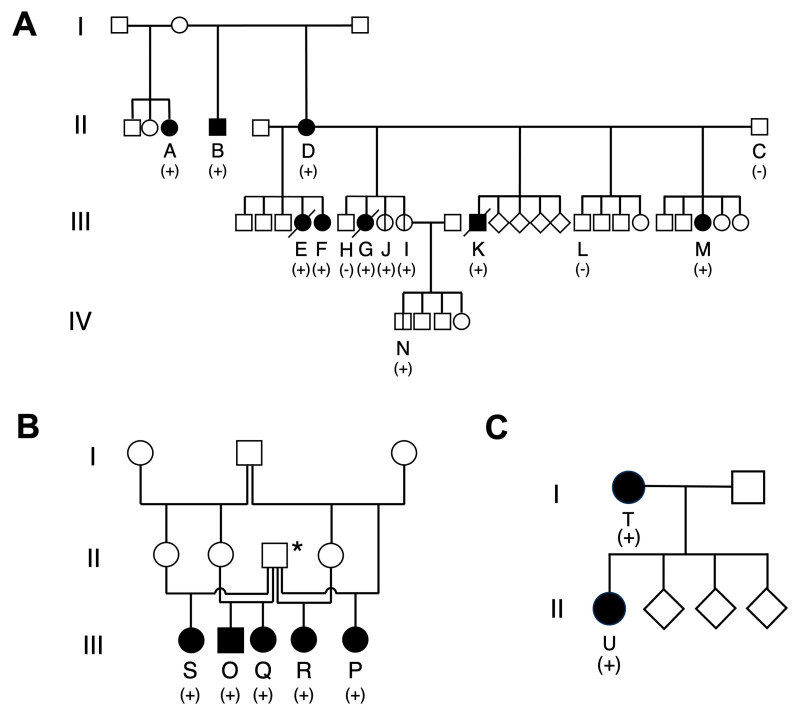
Family trees of families 1, 2, and 3. The examined cases are assigned alphabetical letters in order of their birthday in each family. The square, circle, and diamond indicate the male, female, and unknown sexes, respectively. The black pattern indicates the dogs with a history of gastrointestinal (GI) neoplastic polyp. (+), heterozygous carrier of *APC* variant; (−), non-carrier. (**A**) Family 1. Family tree constructed centering on dog D. Seven offspring and a grandchild (dog N) of dog D harbor the germline *APC* variant. In addition, a full brother (dog B) and a half sister (dog A) of dog D have the variant. In total, 11 dogs have the *APC* variant, of which eight have GI lesions. The diagonal and vertical line indicate the deceased dogs and the dogs without a history of GI neoplastic polyps, respectively. (**B**) Family 2. Family tree of five paternal siblings affected with hereditary GI polyposis. Dogs O and Q are full siblings; the other dogs are their paternal half sisters. The asterisks indicate their common father suspected of having the germline *APC* variant. (**C**) Family 3. Mother–daughter pair.

The first family comprised 14 blood-related JRTs across three generations (Figure 1A and Table 1). All examined dogs, except for dog C, had a blood relationship with dog D.

Initially, a female JRT (dog G) was referred to the Nara Animal Referral Clinic to diagnose and treat GI polyps. One year later, another female JRT (dog E) was referred for the treatment of a rectal polyp and was found to be the maternal half sister of dog G based on information from the dog owners. Genetic testing revealed that both cases harbored the heterozygous germline *APC* variant c.[462_463delinsTT]. Subsequently, we traced their parents based on the information on their certificates. We examined their common dam (dog D) and the sire (dog C) of dog G. As expected, the sire tested negative, and the dam harbored the heterozygous germline *APC* variant, indicating that the affected daughters inherited the variant from their mother. The dam developed rectal adenocarcinoma three years after giving birth to the daughters. Thereafter, with the cooperation of the breeder, we could trace other offspring of the same parents, including three littermates and two non-littermate full siblings of dog G (dogs H–L). Additionally, a younger offspring (dog M) was referred for treatment of rectal prolapse. Among them, four were positive, and two were negative for the germline *APC* variant. One of the female dogs with the *APC* variant (dog I) had already given birth at the time of genetic testing, and one of her pups with the *APC* variant (dog N) was identified in a recent large-scale epidemiological survey [26]. Furthermore, the dam had a litter with another male dog. While the sire was unavailable for evaluation of the *APC* genotype, a littermate full sister (dog F) of dog E was referred for treatment of a rectal polyp and tested positive for the *APC* variant.

During our evaluation of whether the relatives of this family were considered previous cases based on the pedigree certificates [7], a full brother and a half sister of dog D (dogs B and A, respectively) were found.

Among the examined dogs in this family, 11 were carriers of the germline *APC* variant. We examined their medical histories through interviews with dog owners and primary-care veterinarians to verify the association between the germline *APC* variant and GI neoplastic polyposis. The medical histories of the carrier dogs are summarized in Table 1. As expected, while no non-carrier dogs developed GI tumors, eight carriers had a current and/or past history of GI neoplastic polyposis.

These findings demonstrate that hereditary GI polyposis was transmitted in association with the germline *APC* variant through the generations in this family of JRTs.

#### 3.1.2. Family 2

The second family comprised five full and paternal half sibling dogs with the *APC* variant (dogs O–S, Figure 1B), and all dogs had histories of GI polyps (Table 1).

A pair of paternal half sisters (dogs R and S) were found in a 2020 epidemiological survey [26]. Thereafter, dog P was referred to the Nara Animal Referral Clinic for gastric polyps and was found to be a paternal half sister based on the pedigree certificate. Furthermore, we found a pair of full siblings with hereditary GI polyposis (dogs O and Q) among the JRTs who received our medical consultation. While these five dogs were paternal siblings, their mothers had a blood relationship; they were full or half sisters (dams of cases O, Q, R, and S) and had a mother–daughter relationship (dams of cases R and P). Unfortunately, examining the parents for the germline *APC* variant was impossible; however, it was highly likely that the father transmitted the germline *APC* variant to the daughters, although we cannot completely reject the possibility of transmission from the mothers.

#### 3.1.3. Family 3

The third family comprised a mother–daughter pair that the same owner kept (Figure 1C). The dam was referred to Gifu University Veterinary Hospital for recurrent GI polyps and had previously been diagnosed with hereditary GI polyposis based on genetic testing results [7]. Before the initial occurrence of GI polyps at five years of age, the female dog (dog U) had delivered four offspring, including the daughter (dog T), at the age of three years. The daughter was brought to the veterinary hospital at 8 years of age with a gastric polyp, and genetic testing revealed that she had inherited the *APC* variant.

### 3.2. Disease Phenotype of the APC Variant Carriers

#### 3.2.1. Brief Summary of the Medical Histories of the *APC* Variant Carriers

Among the 18 dogs that tested positive for the *APC* variant in the three families, 15 had current and/or previous histories of GI tumors (Table 1). Dog N was strongly suspected of having GI tumors based on severe GI symptoms; however, it was left clinically undiagnosed. In family 1, there were two asymptomatic dogs at the time of the interview with their owners; dogs I and J were asymptomatic at 4.4 and 5 years of age, respectively, and thus were not screened for GI tumors. Despite the presence of these dogs, the germline *APC* variant was significantly associated with GI polyposis in family 1 (*p* < 0.05, Fisher’s exact test, Supplementary Table S1).

In addition to GI tumors, epidermal cysts (*n* = 6; dogs E, F, G, M, P, and U) and mammary adenomas (*n* = 3; dogs F, F, and U) were frequently observed in the examined cases.

#### 3.2.2. Number and Distribution of the GI Lesions

**Figure 2:**
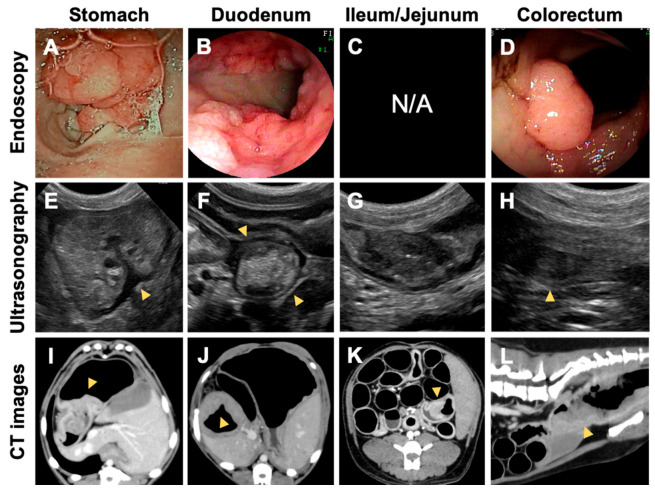
Clinical images of polypoid lesions that developed throughout the gastrointestinal (GI) tract in the Jack Russell terriers carrying the germline *APC* variant. Representative endoscopic (**A**–**D**), ultrasonographic (**E**–**H**), and CT (**I**–**L**) images of the GI lesions. Imaging examinations revealed masses protruding into the lumen of the stomach and rectum and circumferential masses in the duodenum. N/A, not applicable.

Table 1 summarizes the number and location of the GI lesions detected clinically. The mean cumulative number of GI lesions among the 15 affected cases was 7.9 ± 7.7 (mean ± standard deviation). The mean number differed substantially among the families: 12.1 ± 8.5 in family 1 (*n* = 8), 2.8 ± 1.8 in family 2 (*n* = 5), and 3.5 ± 0.7 (*n* = 2) in family 3. While the numbers in families 2 and 3 were comparable with a previously reported number [7], a far greater number of GI tumors were found in family 1, with 27 polyps in dog E being the highest. Consistent with the increased number of GI lesions, the GI polyps were more widely distributed throughout the GI tract than previously reported [7]. Unlike in past cases [7], five cases in family 1 developed multiple tumors in the duodenum and/or jejunum or ileum (Figure 2). Small intestinal tumors were detected in a dog from family 2 (dog Q). In family 1, many cases developed initial GI tumors at a younger age than previous cases [7]. While the average onset age was 7.7 years in previous cases [7], six cases developed initial GI tumors at age < 5 years, three cases at the age of 3 years, and three cases at the age of 4 years. If compared with previous cases [7], the proportion of the dogs with onset at younger ages was significantly higher in family 1 (Table 2). The younger cases, such as dogs G and K, developed a higher number of GI polyps.

#### 3.2.3. Pathological Findings

**Figure 3:**
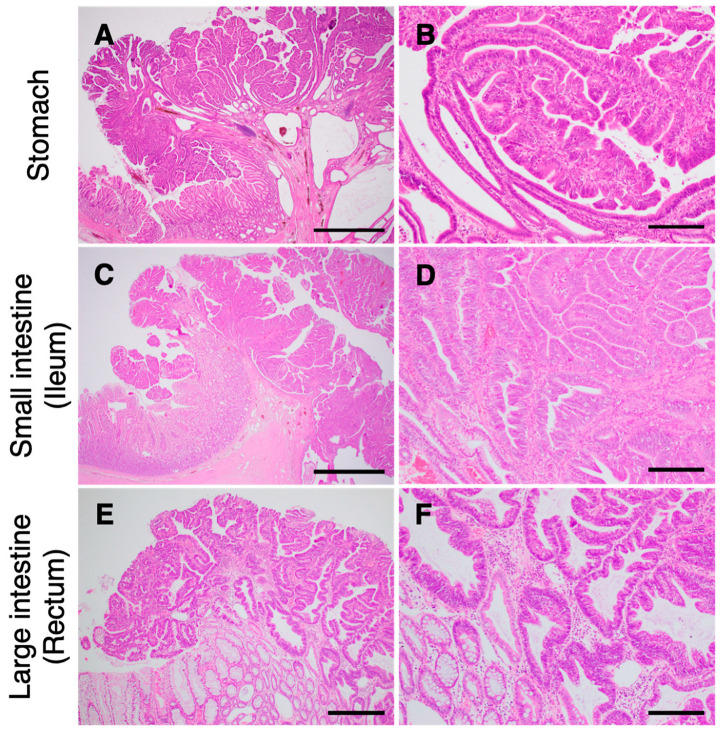
Photomicrographs of gastrointestinal (GI) tumors in the Jack Russell terriers carrying the germline *APC* variant. Representative photomicrographs of adenocarcinomas in the stomach (**A**,**B**), ileum (**C**,**D**), and rectum (**E**,**F**). (**B**,**D**,**F**), higher magnification of (**A**,**C**,**E**), respectively. Tumor cells proliferate mainly in the upper epithelial layer of the GI mucosa. Scale bars are 2 mm (**A**,**E**), 500 µm (**C**), and 200 µm (**B**,**D**,**F**).

In total, 83 GI polyps from 15 JRTs, including 26 gastric, eight small intestinal, and 49 colorectal lesions, were examined histopathologically. The gastric polyps were diagnosed as hyperplastic polyps (*n* = 1), adenomas (*n* = 10), or adenocarcinomas (*n* = 15). All small intestinal tumors (*n* = 8) were diagnosed as adenocarcinomas. Except for a rectal polyp diagnosed as an adenoma using endoscopic biopsy specimens, colorectal polyps (*n* = 48) were adenocarcinomas (Supplementary Table S2). Consistent with a previous report [7], invasion through the muscularis mucosa was not observed for most GI tumors (Figure 3). In contrast, muscle layer invasion was detected in adenocarcinoma of the jejunum in dog G.

## 4. Discussion

In this study, we traced the close members of three unrelated JRT families with the active cooperation of their owners, breeders, and primary-care veterinarians and provided the first evidence that hereditary GI polyposis is transmitted in association with the germline *APC* variant through generations in dogs. The study limitation is that the examined dogs were significantly biased, and it was impossible to calculate the transmission rate. Theoretically, one may believe that the germline *APC* variant is transmitted to approximately 50% of offspring. However, because this study included a high proportion of dogs found in the clinical practice, the variant carriers were detected at a much higher rate than theoretically expected. Additionally, while three dogs in families 1 or 2 were found in the carrier dogs detected in a previous epidemiological study [26], it was impossible to find any relative non-carrier dogs in this study because of their lacking pedigree certificates. In the epidemiological study, the certificates of the non-carrier dogs were not collected [26]. Despite the limitations, this study complements the previous case-control study [7] by providing actual examples of familial transmission of this disease.

In Japan, the number of JRTs with hereditary GI polyposis increased in the late 2000s [7]. In 2020, the overall prevalence of the germline *APC* variant was 1.89 %; the prevalence did not significantly change according to age, suggesting that the prevalence remained flat during the last 15 years [26]. The three JRT families examined in this study illustrate how the germline *APC* variant is carried out and maintained. In family 1, the dam with the germline *APC* variant (dog D) had already given birth to seven litters with 26 offspring between the ages of 1 and 4 years before GI tumors were first detected at 4.6 years, and it transmitted the *APC* variant to many offspring. Similarly, in family 3, the dam delivered four offspring at the age of 3 years before disease onset at 5 years, and at least one daughter inherited the *APC* variant. These examples reveal that unintentional breeding involving germline *APC* variant carriers may occur before the onset of the disease. Meanwhile, in family 2, the sire likely transmitted the germline *APC* variant to his daughters, although genetic testing of the sire was not possible. Based on the birthdays of the daughters, this sire was used for breeding for at least 4.5 years. When a popular sire, such as a dog show champion, is a carrier of disease-associated variants, the variant can spread rapidly within the breed because of the high-value dog’s extensive use for breeding [1]. Although the detailed and accurate breeding history of the sire was unknown, this case may be a typical example of the popular sire effect. These findings emphasize the necessity of breeding programs incorporating genetic screening for the germline *APC* variant, which would substantially reduce the incidence of and eventually eradicate this hereditary disease. In addition, the pedigree analysis revealed that many dogs introduced from Australia, including Australian champion dogs, were present in the ancestors of the three families examined herein; this suggests that there may be blood-related offspring with the germline *APC* variant in other countries.

Our investigation of the medical history of the carrier dogs verified the association between the germline *APC* variant and GI neoplastic polyposis. As expected, most dogs harboring the germline *APC* variant (15/18 dogs) had a current and/or past history of GI tumors. Two dogs remained asymptomatic but did not reach the reported average onset age of 7 years and 8 months [7]. While the penetrance of the germline *APC* variants is thought to be almost 100% in human FAP [27], further follow-up surveys on carriers are required to determine whether the *APC* variant exhibits complete penetrance in JRTs.

Many current cases, especially those in family 1, showed more severe disease phenotypes than previously reported cases [7]. In family 1, the mean number of GI lesions was higher than previously reported. Further, three of the eight dogs with GI polyps in family 1 had more than 10 tumors. In contrast, only one of the 21 affected dogs showed a comparable number [7]. Dogs E, F, and K had more colorectal polyps, while multiple lesions were more frequently observed in the stomach than in the colorectum in previous cases [7]. In addition, three cases in family 1 developed initial GI tumors at 3 years of age, and the other three cases at 4 years, with the dogs being significantly younger than the average onset age of 7 years and 8 months in previous cases [7]. The current cases showed a wider distribution of GI tumors. Previously, the predilection sites for GI polyps were reported to be the stomach and large intestine in JRTs carrying the germline *APC* variant, and only one case had a history of a duodenal lesion among the 21 examined cases [7]. In contrast, six cases in this study developed multiple lesions in the duodenum, jejunum, or ileum, of which four cases in family 1 simultaneously had multiple tumors throughout the GI tract. Among the more severe cases, four died from disease-related causes at relatively young ages, of which two developed systemic metastases (dogs E and G). As in sporadic GI cancers in dogs, hereditary GI polyposis can progress to potentially lethal conditions in rare cases. The presence of severe cases suggests that the severity of the disease can vary considerably among JRTs with hereditary GI polyposis. Additionally, the significantly higher incidence of severe cases in family 1 suggests that genetic modifiers involved in increasing severity may have been transmitted in this family in addition to the germline *APC* variant. This hypothesis is supported by the fact that the severe cases were close relatives; however, they were kept by different owners in different environments. A previous study showed that first-degree relatives had more similar polyp counts than distant relatives in FAP human patients, suggesting that closer relatives share alleles at a modifier locus and thus have more similar phenotypes [28]. A few candidate modifier genes/loci of disease severity have been reported in FAP patients [29,30].

## 5. Conclusions

Our study demonstrated that hereditary GI polyposis was transmitted in association with the germline *APC* variant through the generations in three unrelated families of JRTs. We verified the association between the germline *APC* variant and GI neoplastic polyposis. Careful evaluation of the disease phenotypes of the family members revealed individual variability in the severity of this disease compared with previous reports. Further studies on genetic modifiers of disease severity would lead to novel insights into the molecular mechanisms of GI tumor development in humans and animals.

## Figures and Tables

**Table 1:** Profile and clinical information of the Jack Russell terriers in the three examined families.

Dog No.	Birthday	Sex	*APC* Variant Status	Onset Age ^‡^	Gastro Intestinal Sign	Age at Death	Cumulative Number and Location of Gastrointestinal Polyps				Treatment for Gastrointestinal Polyps	
							Stomach	Duodenum	Jejunum and Ileum	Large Intestine	Surgical Treatment	Medical Treatment
Family 1												
Generation II ^†^												
A	13 April 2010	F	Carrier	6 y 2 m	+		5	0	0	0	+	
B	18 March 2013	M	Carrier	4 y 10 m	+		0	0	0	3	+	
C	18 July 2013	M	Non-carrier		−						−	
D	10 October 2013	F	Carrier	4 y 7 m	+		2	0	2	2	+	
Generation III ^†^												
E	28 October 2014	F	Carrier	4 y 10 m	+	7.6 y ^§^	10	0	2	15	+	NSAID
F	28 October 2014	F	Carrier	7 y 3 m	+		1	0	0	8	+	NSAID
G	29 May 2015	F	Carrier	3 y 2 m	+	6.3 y ^§§^	5	1 *	9	3	+	NSAID, Toceranib
H	29 May 2015	M	Non-carrier		−						−	
I	29 May 2015	F	Carrier		− (at 4.4 years old)		NT	NT	NT	NT	−	
J	29 May 2015	F	Carrier		− (at 5 years old)		NT	NT	NT	NT	−	
K	19 November 2015	M	Carrier	3 y 3 m	+	4.6 y ^§§§^	1	1	7	11	+	NSAID, Toceranib
L	30 June 2016	M	Non-carrier		−						−	
M	11 December 2016	F	Carrier	3 y 10 m	+		0	0	4	5	+	
Generation IV ^†^												
N	30 May 2017	M	Carrier	NT	+		NT	NT	NT	NT	−	
Family 2												
O	3 November 2011	M	Carrier	7 y 3 m	+		5	0	0	0	+	NSAID
P	12 July 2013	F	Carrier	8 y	+		4	0	0	0	+	NSAID
Q	30 September 2013	F	Carrier	6 y 8 m	+		0	1 *	1	1	−	NSAID
R	15 October 2014	F	Carrier	6 y	+		1	0	NT	NT	−	
S	21 January 2017	F	Carrier	3 y 10 m	+		NT	NT	NT	1	+	
Family 3												
T	25 November 2008	F	Carrier	5 y 1 m	+	8.9 y ^§§§§^	2	0	0	1		
U	24 January 2012	F	Carrier	8 y 9 m	+		3	0	0	1		

NT, not tested; NSAID, non-steroidal anti-inflammatory drug; ^†^ generation numbers match those in Figure 1A; ^‡^ age at which the initial gastrointestinal tumors were detected; * circumferential mass; ^§^ case E died from a systemic metastasis of adenocarcinoma of the ileum and/or colon; ^§§^ case G died from a systemic metastasis of adenocarcinoma of the ileum; ^§§§^ case K died from a small intestinal perforation near the tumor; ^§§§§^ case T died from a suspected gastric perforation near the tumor.

**Table 2:** Comparison of onset age of gastrointestinal polyps between Jack Russell terriers in family 1 and previous cases.

	Onset Age of Gastrointestinal Polyps		
	Aged < 5 Years	Aged ≥ 5 Years	*p-*Value *
Family 1 ^†^	6	3	0.01
Previous cases (Ref. [7]) ^‡^	3	17	

^†^ Two dogs without available follow-up data at age 5 years (dogs I and N) were excluded. ^‡^ One dog that was found to be a member of family 1 (dog A) was excluded. * The significance of difference was statistically evaluated using the Fisher’s exact test.

## Data Availability

The data discussed in this study are openly available in the GenBank repository at https://www.ncbi.nlm.nih.gov/genbank/ (accessed on 4 July 2023), reference numbers LC726205–LC72619.

## Supplementary Materials

The following supporting information can be downloaded at: https://www.mdpi.com/article/10.3390/vetsci10070439/s1, Table S1: Intrafamilial analysis on association between the germline APC variant and gastrointestinal polyposis in family 1; Table S2: Histopathological diagnosis of gastrointestinal polyps in the Jack Russell terriers in the three examined families.
