# Supplementary material for: First Evidence of Familial Transmission of Hereditary Gastrointestinal Polyposis Associated with Germline APC Variant in Jack Russell Terriers
Source: Vet Sci. 2023 Jul 5;10(7):439. doi: 10.3390/vetsci10070439 (PMC10385476; doi:10.3390/vetsci10070439)
Supplement: Supplementary file 1 [file vetsci-10-00439-s001.zip › vetsci-2416026-supplementary.pdf]

Supplementary Table S1. Intrafamilial analysis on association between the germline *APC* variant and gastrointestinal polyposis in family 1.

	Gastrointestinal polyposis		
	Affected	Unaffected	*P* value*
APC variant carriers†	8	2	< 0.05
Non-carriers	0	3	

†Dog N that had severe gastrointetinal symptom but was left clinically undiagnosed are excluded.

*The significance of difference was statistically evaluated using the Fisher's exact test.

Supplementary Table S2. Histopathological diagnosis of gastrointestinal polyps in the Jack Russell terriers in the three examined families.

Dog No.	Lesion No.	Location		Age at excision	Method of excision		Histological diagnosis [1]	Histological type [1]	Depth of Invasion (T status) [2]
Family 1									
Generation II									
A	A-G01	Stomach	Antrum	9y1m	S	(Billroth I)	Adenocarcinoma	Tubulopapillary	pTis
B	B-L01	Large intestine	Rectum	4y10m	S	(pull-through)	Adenocarcinoma	ND	ND
	B-L02	Large intestine	Rectum	6y1m	E		Adenocarcinoma	Papillary	pTis
D	D-G01	Stomach	Antrum	5y4m	E		Adenoma	Tubulopapillary	NA
	D-L01	Large intestine	Colon		E		Adenocarcinoma	Papillary	NA
	D-L02		Rectum		S	(pull-through)	Adenocarcinoma	Papillary	pTis
Generation III									
E	E-G01	Stomach	Antrum	4y11m	E		Adenoma	Tubulopapillary	NA
	E-G02		Antrum	6y	S	(local excision)	Adenocarcinoma	Tubulopapillary	pTis
	E-G03		Antrum		S	(local excision)	Adenocarcinoma	Tubulopapillary	pTis
	E-G04		Antrum	6y9m	S	(local excision)	Adenoma	Tubulopapillary	pTis
	E-G05		Antrum		S	(local excision)	Adenocarcinoma	Tubulopapillary	(-)
	E-G06		Antrum		S	(local excision)	Adenocarcinoma	Tubulopapillary	pTis
	E-G07		Antrum		S	(local excision)	Adenocarcinoma	Tubulopapillary	pTis
	E-G08		Antrum		S	(local excision)	Adenocarcinoma	Tubulopapillary	pTis
	E-G09		Antrum		S	(local excision)	Adenocarcinoma	Tubulopapillary	pTis
	E-G10		Antrum		S	(local excision)	Adenocarcinoma	Tubulopapillary	pTis
	E-S01	Small intestine	Jejunum	6y	S	(end-to-end anastomosis)	Adenocarcinoma	Papillary	pTis
	E-S02		Jejunum		S	(end-to-end anastomosis)	Adenocarcinoma	Papillary	pTis
	E-L01	Large intestine	Colon	4y10m	E		Adenocarcinoma	Papillary	NA
	E-L02		Rectum	5y	S	(local excision)	Adenocarcinoma	Papillary	pTis
	E-L03		Rectum	6y1m	S	(pull-through)	Adenocarcinoma	Papillary	pTis
	E-L04		Rectum		S	(pull-through)	Adenocarcinoma	Papillary	pTis
	E-L05		Rectum		S	(pull-through)	Adenocarcinoma	Papillary	pTis
	E-L06		Rectum		S	(pull-through)	Adenocarcinoma	Papillary	pTis
	E-L07		Rectum		S	(pull-through)	Adenocarcinoma	Papillary	pTis
	E-L08		Rectum		S	(pull-through)	Adenocarcinoma	Papillary	pTis
	E-L09		Rectum		S	(pull-through)	Adenocarcinoma	Papillary	pTis
	E-L10		Rectum		S	(pull-through)	Adenocarcinoma	Papillary	pTis
	E-L11		Rectum		S	(pull-through)	Adenocarcinoma	Papillary	pTis
	E-L12		Rectum		S	(pull-through)	Adenocarcinoma	Papillary	(-)
	E-L13		Rectum		S	(pull-through)	Adenocarcinoma	Papillary	(-)
	E-L14		Rectum		S	(pull-through)	Adenocarcinoma	Papillary	(-)
	E-L15		Rectum		S	(pull-through)	Adenocarcinoma	Papillary	(-)
F	F-G01	Stomach	Antrum	7y7m	S	(local excision)	Adenocarcinoma	Tubulopapillary	pTis
	F-L01	Large intestine	Rectum	7y5m	S	(pull-through)	Adenocarcinoma	Papillary	pTis
	F-L02		Rectum		S	(pull-through)	Adenocarcinoma	Papillary	pTis
	F-L03		Rectum		S	(pull-through)	Adenocarcinoma	Papillary	pTis
	F-L04		Rectum		S	(pull-through)	Adenocarcinoma	Papillary	pTis
	F-L05		Rectum		S	(pull-through)	Adenocarcinoma	Papillary	pTis
	F-L06		Rectum		S	(pull-through)	Adenocarcinoma	Papillary	pTis
	F-L07		Rectum		S	(pull-through)	Adenocarcinoma	Papillary	pTis
	F-L08		Rectum		S	(pull-through)	Adenocarcinoma	Papillary	pTis
G	G-G01	Stomach	Antrum	3y6m	E		Adenocarcinoma	Papillary	NA
	G-S01	Small intestine	Duodenum		E		Adenocarcinoma	Papillary	NA
	G-S02		Jejunum/Ilenum	4y7m	S	(end-to-end anastomosis)	Adenocarcinoma	Papillary	pT2
	G-S03		Jejunum/Ilenum		S	(end-to-end anastomosis)	Adenocarcinoma	Papillary	pT1a
	G-S04		Jejunum/Ilenum		S	(end-to-end anastomosis)	Adenocarcinoma	Papillary	pT1a
	G-L01	Large intestine	Rectum	3y4m	S	(local excision)	Adenocarcinoma	Papillary	pTis
	G-L02		Rectum		S	(local excision)	Adenocarcinoma	Papillary	pTis
K	K-G01	Stomach	No data	3y3m	E		Hyperplastic polyp	NA	NA
	K-S01	Small intestine	Duodenum		E		Adenocarcinoma	Papillary	NA
	K-L01	Large intestine	Colon		E		Adenocarcinoma	Papillary	NA
	K-L02		Rectum		E		Adenocarcinoma	Papillary	NA
	K-L03		Colon	4y2m	S	(pull-through)	Adenocarcinoma	Papillary	pTis
	K-L04		Colon		S	(pull-through)	Adenocarcinoma	Papillary	pTis
	K-L05		Colon		S	(pull-through)	Adenocarcinoma	Papillary	pTis
	K-L06		Colon		S	(pull-through)	Adenocarcinoma	Papillary	pTis
	K-L07		Colon		S	(pull-through)	Adenocarcinoma	Papillary	pTis
	K-L08		Rectum		S	(pull-through)	Adenocarcinoma	Papillary	pTis

	K-L09		Rectum		S (pull-through)	Adenocarcinoma	Acinar	pTis
	K-L10		Rectum		S (pull-through)	Adenocarcinoma	Papillary	pTis
	K-L11		Rectum		S (pull-through)	Adenocarcinoma	Papillary	pTis
	K-L12		Rectum		S (pull-through)	Adenocarcinoma	Papillary	pTis
M	M-L01	Large intestine	Rectum	3y11m	S (pull-through)	Adenocarcinoma	Papillary	pT1
	M-L02		Rectum		S (pull-through)	Adenocarcinoma	Papillary	pTis
	M-L03		Rectum		S (pull-through)	Adenocarcinoma	Papillary	pTis
	M-L04		Rectum		S (pull-through)	Adenocarcinoma	Papillary	pTis
Family 2								
O	O-G01	Stomach	Antrum	7y3m	S (local excision)	Adenoma	Papillary	ND
	O-G02		Antrum		S (local excision)	Adenoma	Papillary	ND
	O-G03		Antrum		S (local excision)	Adenoma	Papillary	ND
	O-G04		Antrum		S (local excision)	Adenoma	Papillary	ND
	O-G05		Antrum	10y2m	S (local excision)	Adenoma	Papillary	ND
P	P-G01	Stomach	Antrum	8y2m	S (local excision)	Adenocarcinoma	Tubulopapillary	pTis
	P-G02		Antrum		S (local excision)	Adenocarcinoma	Tubular	pTis
	P-G03		Antrum		S (local excision)	Adenocarcinoma	Tubular	pTis
Q	Q-S01	Small intestine	Duodenum	6y9m	E	Adenocarcinoma	Papillary	NA
	Q-L01	Large intestine	Colon		E	Adenocarcinoma	Papillary	NA
R	R-G01	Stomach	Antrum	6y2m	E	Adenocarcinoma	Tubulopapillary	NA
S	S-L01	Large intestine	Colon	3y10m	S (unknown)	Adenocarcinoma	ND	ND
Family 3								
T	T-G01	Stomach	Cardia	8y9m	E	Adenoma	Tubulopapillary	NA
	T-L01	Large intestine	Colon		E	Adenocarcinoma	Papillary	NA
U	U-G01	Stomach	Antrum	8y9m	E	Adenoma	Tubulopapillary	ND
	U-L01	Large intestine	Rectum	10y9m	E	Adenoma	ND	NA

S, surgical resection; E, endoscopic biopsy; ND, no data are available from pathological reports of other laboratories; NA: not applicable; (-), not evaluated because of the absence of submucosa and deeper tissues.

[1] Histological Classification of Tumours of the Alimentary System of Domestic Animals (WHO International Classification of Tumors of Domestic Animal Series) (Head KW et al., 2003).

[2] The depth of invasion of GI adenocarcinomas was evaluated according to the T stage criteria in the TNM classification as follows (TNM classification of malignant tumors, 8th edition, 2017).
Stomach Tis: Intraepithelial tumor without invasion of the lamina propria (carcinoma in situ), T1a: Tumor invades lamina propria or muscularis mucosae, T1b: Tumor invades submucosa, T2 Tumor invades muscularis propria, T3: Tumor invades subserosa, T4a: Tumor perforates serosa (visceral peritoneum), T4b: Tumor invades adjacent structures.
Small intestine Tis: Carcinoma in situ, T1a: Tumor invades lamina propria or muscularis mucosae, T1b: Tumor invades submucosa, T2: Tumor invades muscularis propria, T3: Tumor invades subserosa or nonperitonealized perimuscular tissue (mesentery or retroperitoneum) with extension 2 cm or less, T4: Tumor perforates visceral peritoneum or directly invades other organs or structure.
Large intestine Tis: Intraepithelial or invasion of lamina propria (carcinoma in situ), T1: Tumor invades submucosa, T2: Tumor invades muscularis propria, T3: Tumor invades subserosa or into non-peritonealized pericolic or perirectal tissue, T4a: Tumor perforates visceral peritoneum, T4b: Tumor directly invades other organs or structure.
